# Validity assessment of the symptom checklist SCL-90-R and shortened versions for the general population in Ukraine

**DOI:** 10.1186/s12888-016-1014-3

**Published:** 2016-08-26

**Authors:** Yuliia Sereda, Serhii Dembitskyi

**Affiliations:** 1 Senior Scientific Associate, Department for Monitoring of Social and Economic Transformations, Institute for Economics and Forecasting, National Academy of Sciences, Kiev, Ukraine; 2 Senior Scientific Associate, Department of Methodology and Methods of Sociology, Institute of Sociology, National Academy of Sciences, Kiev, Ukraine

**Keywords:** SCL-90-R, Short versions, Mental disorders, Symptomatic distress, Self-report questionnaire

## Abstract

**Background:**

The Symptom Checklist-90-Revised (SCL-90-R) is a widely used symptomatic distress questionnaire. A translated version of the SCL-90-R has been applied in Ukrainian general population surveys several times but has not yet been validated in this country. The SCL-90-R and its short versions (BSI-53, SCL-27, BSI-18, SCL-14 and SCL-9-K) were investigated in order to comparatively assess their properties and applications in Ukraine.

**Methods:**

Secondary analysis of three nationally representative cross-sectional surveys (1997, 1999 and 2014) using SCL-90-R was applied. Two thousand sixty nine respondents participated in 2014; the sample size for the 1997 and 1999 surveys was 1810 respondents per wave. Statistical data analysis is based on calculating internal consistencies with Cronbach’s Alpha, confirmatory factor analysis, nonparametric correlations and effect sizes for the equivalence of the full and short versions.

**Results:**

The scales of SCL-90-R and its shortened versions showed equally high internal consistencies. With regard to factorial validity, 2014 data confirmed the dimensional structure of all versions. Unsatisfactory results were found in 1997 and 1999 for SCL-90-R and in 1997 for SCL-27, based on the Chi-square criterion (*χ*2/degrees of freedom > 5), though other indexes suggested satisfactory model fit (RMSEA < 0.06; CFI, TLI > 0.95). Analysis of the equivalence of shortened and full versions of the SCL-90-R has shown the presence of small effect sizes.

**Conclusion:**

BSI-18 and SCL-9-K are recommended for use in general population surveys as more economical versions of SCL-90-R. Both versions revealed satisfactory validity in 1997, 1999 and 2014.

**Electronic supplementary material:**

The online version of this article (doi:10.1186/s12888-016-1014-3) contains supplementary material, which is available to authorized users.

## Background

Symptom Checklist-90-Revised (SCL-90-R) is a widely used questionnaire developed by Leonard R. Derogatis [1] to determine a number of psychological symptoms. In Ukraine, SCL-90-R was first used in the study “Mental health of children after the Chernobyl disaster” [2]. Later, it was applied in three surveys with samples that were representative of the entire population (1997, 1999 and 2014), but it has not yet been validated.

SCL-90-R includes 90 symptoms and evaluates nine symptomatic dimensions: somatization, obsessive-compulsive disorder, interpersonal sensitivity, depression, anxiety, hostility, phobic anxiety, paranoid ideation, and psychoticism [1]. Given the demand for briefer measures to be used as a screening tool for psychiatric disorders, shortened versions of SCL-90-R were developed, such as BSI-53 [3], SCL-27 [4], BSI-18 [5], SCL-14 [6, 7] and SCL-9-K [6, 8]. BSI-53 includes all nine symptomatic dimensions with a reduced number of symptoms, whereas SCL-27, BSI-18 and SCL-14 have both reduced factor structures and reduced numbers of items. SCL-9-K is the shortest measure, including nine symptoms within a single dimession (general severity factor). Numbers of indicators for symptomatic dimensions in SCL-90-R and its shortened versions are presented in Table 1.

**Table 1:** Dimensional structure and items of the SCL-90-R and its shortened versions

SCL-90-R		BSI-53		SCL-27		BSI-18		SCL-14		SCL-K-9	
Scale	Indicators	Scale	Indicators	Scale	Indicators	Scale	Indicators	Scale	Indicators	Scale	Indicators
SOMA	1, 4, 12, 27, 40, 42, 48, 49, 52, 53, 56, 58	SOMA	4, 12, 40, 48, 49, 52, 56	VEG	4, 39, 40, 48, 49, 53	SOMA	12, 40, 48, 52, 56, 58	VEG	42, 52, 56, 58		
OCD	3, 9, 10, 28, 38, 45, 46, 51, 55, 65	OCD	9, 28, 45, 46, 51, 55	DYS	9, 14, 51, 55						
INT	6, 21, 34, 36, 37, 41, 61, 69, 73	INT	34, 37, 41, 69	SOP	37, 41, 61, 69						
DEPR	5, 14, 15, 20, 22, 26, 29, 30, 31, 32, 54, 71, 79	DEPR	15, 29, 30, 32, 54, 79	DEP	15, 30, 54, 59	DEPR	15, 29, 30, 32, 54, 79	DEP	26, 28, 30, 54, 77, 79		
ANX	2, 17, 23, 33, 39, 57, 72, 78, 80, 86	ANX	2, 23, 33, 57, 72, 78			ANX	2, 33, 57, 72, 78, 86				
HOST	11, 24, 63, 67, 74, 81	HOST	11, 24, 63, 67, 74								
PHOB	13, 25, 47, 50, 70, 75, 82	PHOB	13, 47, 50, 70, 75	AGO	13, 25, 33, 50, 82			AGO	13, 25, 47, 82		
PARA	8, 18, 43, 68, 76, 83	PARA	8, 18, 43, 76, 83	MIS	18, 68, 76, 83						
PSYC	7, 16, 35, 62, 77, 84, 85, 87, 88, 90	PSYC	7, 77, 85, 88, 90								
ADD	19, 44, 59, 60, 64, 66, 89	ADD	19, 44, 59, 89								
GSI	all above	GSI	all above	GSI	all above	GSI	all above	GSI	all above	GSI	24, 28, 31, 34, 43, 57, 58, 75, 77

*SOMA* Somatization, *OCD* Obsessive-Compulsive Disorder, *INT* Interpersonal Sensitivity, *DEPR* Depression, *ANX* Anxiety, *HOST* Hostility, *PHOB* Phobic Anxiety, *PARA* Paranoid Ideation, *PSYC* Psychoticism, *GSI* Global Severity Index, *DEP* Depressive Symptoms, *DYS* Dysthymic Symptoms, *VEG* Vegetative Symptoms, *AGO* Agoraphobic Symptoms, *SOP* Symptoms of Social Phobia, *MIS* Symptoms of Mistrust

The vast majority of psychometric studies studies on SCL-90-R were conducted on clinical samples, such as patients of mental health centers and agencies [9, 10], patients with depression [11], patients undergoing personality-centered therapy [12], forcibly hospitalized patients with mental disorders [13], adults and adolescents hospitalized with crisis intervention [14], substance abusers [15], patients with panic disorders [16], veterans undergoing psychiatric treatment [17], patients waiting for bariatric surgery [18], volunteers for drug trials [19] etc. A number of studies estimated properties of SCL-90-R on non-clinical samples, in particular those representative of the entire population or of certain communities; such studies were conducted in Canada [20], Denmark [21], Finland [22], Germany [23], Hungary [24], Japan [25], Italy [26], Norway [27], Thailand [28] and the USA [29].

Overall, there is increasing agreement on the multidimensional nature of the SCL-90-R, although various solutions from bifactor structure [24] to the nine original dimesions [16, 22, 23, 25] have been reported. A few studies support the unidimensional structure of the SCL-90-R as broad construct of distress [21, 28]. Weakness of the validity of SCL-90-R is explained by different reasons, including limitations of sample design and statistical measures. A German study revealed that subscale internal reliabilities are better for clinical samples when compared to non-clinical samples, which might result in revision of the SCL-90-R for the general population [23]. R. Urbán et al. [24] highlighted that the vast majority of studies inappropriately used methods considering responses on a linear scale instead of an ordinal scale, and implemented the maximum likelihood estimator for measuring factor validity, which underestimates the fit of the models in confirmatory factor analysis, resulting in weak structural validity.

Comparative validation of the SCL-90-R and its shortened versions requires further investigation. While the vast majority of papers focus on the full version of the SCL-90-R, Müller et al. [30] examined the validity of eleven shortened versions and recommended SCL-10S as an instrument to measure psychological distress. Recently, Prinz et al. compared the psychometric properties of five shortened versions and concluded that BSI-18 appears to be the most economical variant and most clinically meaningful instrument [6]. None of the comparative validation studies of the SCL-90-R were conducted on non-clinical samples.

Given that previous studies did not come up with a single solution regarding factor validity of the SCL-90-R, we attempt to investigate SCL-90-R in order to comparatively assess its properties and application in Ukraine. Moreover, we concentrate on the comparative validation of SCL-90-R and its five shortened versions (BSI-53, SCL-27, BSI-18, SCL-14, SCL-9-K) in order to assess the extent to which they can reliably measure psychological distress as well as certain distress subscales. In particular, we examine which shortened version provides superior reliability, validity and practical utility in national monitoring surveys with representative samples. Our choice of shortened versions is driven by the evidence that BSI-53 and SCL-27 showed superior discriminant validity while BSI-18, SCL-14 and SCL-9-K demonstrated better performance regarding the general severity factor among the shortest versions in the previous studies [6, 30].

## Methods

### Design

The research is based on a secondary analysis of data collected by the Institute of Social Sciences, National Academy of Sciences of Ukraine (a social monitoring “Ukrainian Society” for 1997 and 1999, principal investigator Prof. Dr. Evgeniy Golovakha), as well as the joint monitoring of the Ukrainian Institute for Social Research after A. Yaremenko, Social Monitoring Center and the Department for Monitoring of Social and Economic Transformations, Institute for Economics and Forecasting, National Academy of Sciences of Ukraine (2014 study, principal investigator Olga Balakireva). In 1997 and 1999, 1810 respondents were interviewed; 2069 respondents were interviewed in 2014. Each of the three cross-sectional studies is representative of the main socio-demographic characteristics of the adult population of Ukraine. In the 1997 and 1999 arrays the sex ratio was 45 % male and 55 % female, and the mean age was 45 years; in the 2014 array, 44 % were male and 56 % were female, and the mean age was 46 years. The 2014 study included 24 regions of Ukraine and Kiev, while in 1997 and 1999, 24 regions of Ukraine, Kiev and the Crimea were included. In all three studies data collection was administered through a face-to-face questionnaire. SCL-90-R was first translated and adapted for Ukraine by Dr. Nataliia Panina for a survey of mothers evacuated from Pripyat, Chernobyl in 1986 [2]. The adequacy of the Ukrainian and Russian translation to the English version was assessed through a back-translation by a professional translator.

### Tools

In all three studies, the questionnaire SCL-90-R was completed as one section of a general questionnaire that included a wide range of social, political and economic aspects. The questionnaire was translated into both Ukrainian and Russian, as different languages were used for different regions of the country. Shortened versions of the symptomatic questionnaire (BSI-53, SCL-27, BSI-18, SCL-14, SCL-9-K) were calculated on the basis of SCL-90-R questions during the secondary analysis.

### Statistical analysis

Included reliability assessment of SCL-90-R subscales, factorial validity of symptomatic measurements and equivalence of individual variants of SCL-90-R. All methods were applied for all three studies (1997, 1999 and 2014).

To assess the reliability of individual symptomatic measures and the Global Severity Index (GSI) in all six versions (SCL-90-R, BSI-53, SCL-27, BSI-18, SCL-14, SCL-9-K) Cronbach’s alpha coefficients were calculated. Values of the coefficient that were higher than 0.7 were considered acceptable [31].

To confirm the factor validity of symptomatic measurements of the full and abbreviated versions of SCL-90-R, confirmatory factor analysis (CFA) was carried out. Given that all indicators have ordinal scales, a Diagonally Weighted Least Squares method (DWLS) was used to estimate the parameters of the CFA, which allows estimation of robust standard errors and correction of the test statistics. Missing values (up to 5 %) were excluded. To assess the quality of the factor models the following indices have been estimated: *χ*2 (Minimum Function Chi-square), RMSEA (The Root Mean Square Error of Approximation), CFI (Comparative fit index) and TLI (Tucker-Lewis index). An acceptable model fit was considered *χ*2/degrees of freedom < 5; RMSEA < 0.06; and CFI, TLI > 0.95 [32].

Since the distribution of all indicators of symptomatic measurements and GSI in the full and shortened versions of the SCL-90-R deviated from normal, nonparametric methods were used for the analysis of equivalence. To analyze the equivalence of the full and shortened versions of SCL-90-R, median and interquartile distances were estimated, the statistical significance of the median differences was calculated on the basis of the Wilcoxon median test, and effect sizes and Spearman’s Rho correlations were defined. We used Vargha and Delaney’s A effect sizes, according to which a small effect is over 0.56; a medium effect is over 0.64, and a large effect is over 0.71 [33]. The size of the correlations was based on the following interpretation limits: rho < 0.30, small correlation; 0.30 > rho < 0.50, medium correlation and rho > 0.50, large correlation [34].

The equivalence of different versions of the SCL-90-R was also evaluated in the context of the size difference of the group with a high risk of psychological distress in the general population, depending on the method, or in other words, the extent to which the prevalence of “probable cases” differs. According to Derogatis’ criterion for the general population, if the GSI has a *T*-value ≥ 63, such individuals may be characterized by the presence of severe symptoms of distress [35]. It is also common to use the criterion of GSI > 1 to determine the proportion of people with severe symptoms of distress [36].

R (package «lavaan» for CFA) and SPSS, version 20 were used for the statistical analysis.

## Results

### Reliability

In the 1997 study Cronbach’s alpha coefficients for different symptomatic measurements ranged from 0.59 (depressive symptoms in SCL-27) to 0.96 (GSI in the SCL-90-R); in 1999 - from 0.63 (depressive symptoms in SCL-27) to 0.97 (GSI in the SCL-90-R), and in 2014 - from 0.66 (depressive symptoms in SCL-27) to 0.98 (GSI in SCL- 90-R) (see Table 2). Cronbach’s alpha coefficients of below an acceptable level of reliability were observed for interpersonal sensitivity in BSI-53 and symptoms of social phobia in the SCL-27 (1997 and 1999), for hostility and phobic anxiety in the 1997 study (in BSI-53), for psychoticism in 1997 and 1999 (in BSI-53), for symptoms of mistrust in 1997 and 1999 (in SCL-27) and for agoraphobic symptoms in 1997 and 1999 (in SCL-14). However, in the 2014 study, the only symptomatic dimension with an unsatisfactory level of reliability was a depressive symptoms scale in SCL-27.

**Table 2:** Reliability of the SCL-90-R subscales and the shortened versions BSI, SCL-27, BSI-18, SCL-14 and SCL-K-9 in the Ukrainian general population

SCL-90-R					BSI-53					SCL-27					BSI-18					SCL-14					SCL-K-9				
Scale	Cronbach’s alpha			Number of items	Scale	Cronbach’s alpha			Number of items	Scale	Cronbach’s alpha			Number of items	Scale	Cronbach’s alpha			Number of items	Scale	Cronbach’s alpha			Number of items	Scale	Cronbach’s alpha			Number of items
	1997	1999	2014			1997	1999	2014			1997	1999	2014			1997	1999	2014			1997	1999	2014			1997	1999	2014	
SOMA	0.90	0.89	0.93	12	SOMA	0.85	0.84	0.89	7	VEG	0.80	0.80	0.87	6	SOMA	0.84	0.84	0.89	6	VEG	0.85	0.84	0.88	4					
OCD	0.81	0.83	0.90	10	OCD	0.76	0.79	0.86	6	DYS	0.74	0.78	0.81	4															
INT	0.80	0.82	0.87	9	INT	0.62	0.66	0.77	4	SOP	0.64	0.69	0.76	4															
DEPR	0.83	0.85	0.91	13	DEPR	0.73	0.75	0.82	6	DEP	0.59	0.63	0.66	4	DEPR	0.73	0.75	0.82	6	DEP	0.72	0.73	0.86	6					
ANX	0.81	0.86	0.92	10	ANX	0.74	0.80	0.87	6						ANX	0.73	0.79	0.86	6										
HOST	0.74	0.75	0.82	6	HOST	0.69	0.70	0.78	5																				
PHOB	0.74	0.78	0.87	7	PHOB	0.68	0.71	0.82	5	AGO	0.71	0.75	0.83	5						AGO	0.64	0.68	0.81	4					
PARA	0.74	0.75	0.84	6	PARA	0.72	0.72	0.81	5	MIS	0.64	0.69	0.76	4															
PSYC	0.79	0.78	0.90	10	PSYC	0.68	0.65	0.82	5																				
GSI	0.96	0.97	0.98	83	GSI	0.94	0.95	0.97	49	GSI	0.90	0.92	0.95	27	GSI	0.88	0.90	0.94	18	GSI	0.84	0.85	0.92	14	GSI	0.81	0.85	0.89	9

*SOMA* Somatization, *OCD* Obsessive-Compulsive Disorder, *INT* Interpersonal Sensitivity, *DEPR* Depression, *ANX* Anxiety, *HOST* Hostility, *PHOB* Phobic Anxiety, *PARA* Paranoid Ideation, *PSYC* Psychoticism, *GSI* Global Severity Index, *DEP* Depressive Symptoms, *DYS* Dysthymic Symptoms, *VEG* Vegetative Symptoms, *AGO* Agoraphobic Symptoms, *SOP* Symptoms of Social Phobia, *MIS* Symptoms of Mistrust

It should be noted that the GSI in different versions of SCL-90-R ranged from 0.81 to 0.98, indicating good reliability. In general, we can note satisfactory reliability for all versions of the symptomatic checklist.

### Factorial validity

According to the RMSEA criteria < 0.06 and CFI, TLI > 0.95, all models have demonstrated satisfactory validity: the results of confirmatory factor analysis generally support the internal structure of symptomatic measures in the SCL-90-R (nine factors), BSI-53 (nine factors), SCL-27 (six factors), BSI-18 (three factors), SCL-14 (three factors) and SCL-90-R (a single factor) (Table 3). A comparison of models fit lower RMSEA and higher CFI and TLI in 2014 compared to the 1997 and 1999 studies. A three-factor model SCL-14 and ten-factor model BSI-53 in the 2014 study are characterized by the lowest *χ*2/DF ratios and the highest CFI and TLI values compared to other models; i.e. these versions of the symptomatic questionnaire demonstrate the best factorial validity.

**Table 3:** Factor validity of SCL-90-R, BSI-53, SCL-27, BSI-18, SCL-14 and SCL-9-K in the Ukrainian general population: confirmatory factor analysis fit

Fit	SCL-90-R			BSI-53			SCL-27			BSI-18			SCL-14			SCL-K-9		
	1997	1999	2014	1997	1999	2014	1997	1999	2014	1997	1999	2014	1997	1999	2014	1997	1999	2014
*χ*2 (DWLS)	25299	21196	15672	6323	6117	4714	1844	1258	1243	575	530	543	415	279	183	111	118	121
DF	3870	3870	3870	1280	1280	1280	309	309	309	132	132	132	74	74	74	27	27	27
*χ*2 / DF	6.5	5.5	4.0	4.9	4.8	3.7	6.0	4.1	4.0	4.4	4.0	4.1	5.6	3.8	2.5	4.1	4.4	4.5
*p*-value	0.000	0.000	0.000	0.000	0.000	0.000	0.000	0.000	0.000	0.000	0.000	0.000	0.000	0.000	0.000	0.000	0.000	0.000
RMSEA (< 0,06)	0.057	0.052	0.042	0.048	0.047	0.038	0.053	0.042	0.040	0.044	0.041	0.040	0.051	0.040	0.027	0.042	0.044	0.042
CFI (> 0,95)	0.967	0.979	0.995	0.980	0.984	0.996	0.977	0.989	0.996	0.991	0.993	0.997	0.988	0.993	0.999	0.993	0.995	0.997
TLI (> 0,95)	0.966	0.978	0.995	0.979	0.983	0.996	0.974	0.987	0.996	0.990	0.992	0.997	0.985	0.991	0.998	0.991	0.993	0.997

*χ*2 (DWLS) = Minimum Function Chi-square for Diagonally Weighted Least Squares

*DF* degrees of freedom, *RMSEA* Root Mean Square Error of Approximation, *CFI* Comparative fit index, *TLI* Tucker-Lewis index

In the 1997 study, the model fit of SCL-14 according to the *χ*2/DF ratio criterion was outside the acceptable limit (*χ*2/DF = 5,6). According to this criterion, poor model fit was also recorded in 1997 and 1999 with respect to SCL-90-R (*χ*2/DF = 6,5 in 1997; *χ*2 / DF = 5,5 in 1999), and with respect to SCL-27 (*χ*2 / DF = 5,6 in 1997). Satisfactory model fit based on all criteria (*χ*2/DF < 5; RMSEA < 0.06 and CFI, TLI > 0.95) and during all three time periods characterizes BSI-53, BSI-18, and SCL-K-9.

Analysis of factor loadings on certain indicators of latent factors – symptomatic measures confirms satisfactory internal consistency (see Additional files 1, 2, 3, 4, 5 and 6 for details). The values of factor loadings in both the shortened and the full version of SCL-90-R exceeded 0.50, except for the indicator “nervousness” in the depression subscale in the SCL-90-R (1997 and 1999 studies). All factor loadings were statistically significant at the 1 % level. There were strong correlations among all latent factors (see Additional files 7, 8, 9, 10 and 11 for details). For example, in the 2014 factor model of SCL-90-R the minimum and maximum correlation of certain symptomatic dimensions is 0.74 (somatization and hostility) and 0.99 (interpersonal sensitivity and paranoid tendencies); in BSI-53 - 0.78 (somatization and hostility, somatization and paranoid tendencies) and 0.99 (interpersonal sensitivity and phobic anxiety); in the SCL-27 - 0.78 (autonomic dysfunction and suspicion) and 0.98 (agoraphobia and social phobia); in BSI-18 - 0.86 (somatization and depression) and 0.98 (depression and anxiety); in the SCL-14 - 0.71 (autonomic disorder and agoraphobia) and 0.87 (agoraphobia and depression).

### Equivalence of the full and shortened versions of the SCL-90-R

Correlations between the full and shortened versions of the SCL-90-R are expectedly very high. Spearman’s Rho correlation coefficients vary in the range of 0.7-0.9 (Table 4). However, if we look at the difference in median values between similar components of SCL-90-R on the one hand, and BSI-53, SCL-27, BSI-18, SCL-14 and SCL-9-K on the other, it appears that in almost all cases, the difference in medians is statistically significant at the 5 % level. Equivalence of medians between full and shortened versions of the questionnaire in all three analyzed studies is observed only for the somatization subscale in the BSI-18. It is noticeable that similar subscales in different versions differ not so much in a measure of central tendency, but in variance. Such differences cause statistically significant differences in small effect sizes. Analysis of the size effects (the so-called “scientific significance”) shows that in all three studies the difference between symptomatic measures of SCL-90-R and BSI-53 is not significant (Vargha and Delaney’s A ≤ 0,56). When comparing SCL-90-R and SCL-27, there is a small effect size for somatization and interpersonal sensitivity (0.56 < k ≤ 0,64, in 1997, 1999 and 2014), whereas effect sizes on other symptomatic dimensions are insignificant. With regard to the comparison of SCL-90-R and such shortened versions as BSI-18, SCL-14, and SCL-9-R, in all three studies minor effect sizes were found, which indicates good equivalence.

**Table 4:** Medians (M) and interquartile ranges (IQR) of the SCL-90-R and the shortened versions: BSI-53, SCL-27, BSI-18, SCL-14, SCL-K-9 and the results of the Wilcoxon signed-rank test (W (p-value)), Vargha and Delaney’s A effect sizes (ES) and Spearman’s correlations (S.Rho) in the general population of Ukraine

1997									1999									2014								
	SCL-90-R			BSI-53						SCL-90-R			BSI-53						SCL-90-R			BSI-53				
Scale	M	IQR	Scale	M	IQR	W (*p*-value)	ES (VD.A)	Rho	Scale	M	IQR	Scale	M	IQR	W (*p*-value)	ES (VD.A)	Rho	Scale	M	IQR	Scale	M	IQR	W (*p*-value)	ES (VD.A)	Rho
SOMA	0.67	0.92	SOMA	0.57	1.00	0.008	0.56	0.95	SOMA	0.67	0.92	SOMA	0.57	1.00	0.013	0.55	0.95	SOMA	0.58	1.00	SOMA	0.43	1.00	0.002	0.56	0.95
OCD	0.40	0.60	OCD	0.33	0.83	<0.001	0.51	0.93	OCD	0.40	0.70	OCD	0.33	0.67	<0.001	0.52	0.93	OCD	0.40	0.80	OCD	0.33	1.00	<0.001	0.50	0.95
INT	0.44	0.67	INT	0.25	0.75	0.729	0.54	0.89	INT	0.44	0.67	INT	0.25	0.75	0.380	0.54	0.90	INT	0.33	0.67	INT	0.25	0.75	<0.001	0.53	0.91
DEPR	0.38	0.62	DEPR	0.33	0.67	<0.001	0.51	0.90	DEPR	0.46	0.69	DEPR	0.50	0.67	<0.001	0.50	0.91	DEPR	0.38	0.77	DEPR	0.33	0.83	<0.001	0.51	0.93
ANX	0.40	0.60	ANX	0.33	0.67	<0.001	0.51	0.93	ANX	0.40	0.60	ANX	0.33	0.50	<0.001	0.49	0.94	ANX	0.30	0.70	ANX	0.33	0.67	<0.001	0.50	0.95
HOST	0.33	0.50	HOST	0.40	0.60	<0.001	0.47	0.98	HOST	0.33	0.50	HOST	0.40	0.60	<0.001	0.47	0.99	HOST	0.33	0.67	HOST	0.40	0.80	<0.001	0.47	0.99
PHOB	0.14	0.29	PHOB	0.00	0.40	0.034	0.50	0.94	PHOB	0.14	0.29	PHOB	0.00	0.40	<0.001	0.49	0.97	PHOB	0.00	0.43	PHOB	0.00	0.40	<0.001	0.50	0.97
PARA	0.50	0.67	PARA	0.40	0.80	0.001	0.47	0.99	PARA	0.50	0.67	PARA	0.40	0.60	0.022	0.48	0.99	PARA	0.33	0.83	PARA	0.40	1.00	<0.001	0.48	0.99
PSYC	0.10	0.40	PSYC	0.00	0.40	<0.001	0.51	0.91	PSYC	0.10	0.40	PSYC	0.20	0.40	<0.001	0.50	0.92	PSYC	0.10	0.44	PSYC	0.00	0.40	<0.001	0.50	0.94
ADD	0.43	0.57	ADD	0.38	0.75	0.221	0.51	0.91	ADD	0.29	0.57	ADD	0.25	0.75	<0.001	0.51	0.92	ADD	0.43	0.71	ADD	0.25	0.75	0.088	0.52	0.92
GSI	0.41	0.53	GSI	0.41	0.55	<0.001	0.51	0.99	GSI	0.42	0.51	GSI	0.41	0.51	<0.001	0.50	0.99	GSI	0.35	0.60	GSI	0.35	0.61	<0.001	0.50	0.99
	SCL-90-R			SCL-27						SCL-90-R			SCL-27						SCL-90-R			SCL-27				
SOMA	0.67	0.92	VEG	0.50	1.00	<0.001	0.60	0.89	SOMA	0.67	0.92	VEG	0.50	0.75	<0.001	0.61	0.89	SOMA	0.58	1.00	VEG	0.25	0.75	0.010	0.59	0.90
OCD	0.40	0.60	DYS	0.25	0.75	0.567	0.53	0.77	OCD	0.40	0.70	DYS	0.50	1.00	<0.001	0.52	0.80	OCD	0.40	0.80	DYS	0.50	1.00	<0.001	0.50	0.86
INT	0.44	0.67	SOP	0.50	0.83	<0.001	0.58	0.86	INT	0.44	0.67	SOP	0.33	0.83	<0.001	0.58	0.87	INT	0.33	0.67	SOP	0.33	1.00	0.010	0.57	0.88
DEPR	0.38	0.62	DEP	0.00	0.40	<0.001	0.50	0.81	DEPR	0.46	0.69	DEP	0.00	0.40	<0.001	0.50	0.83	DEPR	0.38	0.77	DEP	0.00	0.60	0.076	0.52	0.87
PHOB	0.14	0.29	AGO	0.25	0.75	0.001	0.51	0.84	PHOB	0.14	0.29	AGO	0.25	0.75	<0.001	0.50	0.84	PHOB	0.00	0.43	AGO	0.25	0.75	<0.001	0.48	0.85
PARA	0.50	0.67	MIS	0.50	1.00	<0.001	0.49	0.95	PARA	0.50	0.67	MIS	0.50	1.00	0.042	0.49	0.94	PARA	0.33	0.83	MIS	0.50	1.00	<0.001	0.49	0.96
GSI	0.41	0.53	GSI	0.41	0.56	<0.001	0.51	0.95	GSI	0.42	0.51	GSI	0.41	0.56	<0.001	0.51	0.96	GSI	0.35	0.60	GSI	0.35	0.63	<0.001	0.51	0.97
	SCL-90-R			BSI 18						SCL-90-R			BSI 18						SCL-90-R			BSI 18				
SOMA	0.67	0.92	SOM	0.50	1.00	0.113	0.54	0.94	SOMA	0.67	0.92	SOM	0.50	1.00	0.190	0.54	0.94	SOMA	0.58	1.00	SOM	0.33	1.00	0.170	0.56	0.94
DEPR	0.38	0.62	DEP	0.33	0.67	<0.001	0.51	0.90	DEPR	0.46	0.69	DEP	0.50	0.67	<0.001	0.50	0.91	DEPR	0.38	0.77	DEP	0.33	0.83	<0.001	0.51	0.93
ANX	0.40	0.60	ANX	0.33	0.50	<0.001	0.51	0.93	ANX	0.40	0.60	ANX	0.33	0.50	<0.001	0.50	0.94	ANX	0.30	0.70	ANX	0.33	0.67	<0.001	0.50	0.95
GSI	0.41	0.53	GSI	0.50	0.67	<0.001	0.46	0.92	GSI	0.42	0.51	GSI	0.50	0.67	<0.001	0.49	0.93	GSI	0.35	0.60	GSI	0.39	0.72	<0.001	0.48	0.94
	SCL-90-R			SCL-14						SCL-90-R			SCL-14						SCL-90-R			SCL-14				
SOMA	0.67	0.92	SOM	0.75	1.25	<0.001	0.52	0.89	SOMA	0.67	0.92	SOM	0.75	1.00	<0.001	0.51	0.90	SOMA	0.58	1.00	SOM	0.50	1.25	<0.001	0.53	0.91
DEPR	0.38	0.62	DEP	0.50	0.67	<0.001	0.48	0.88	DEPR	0.46	0.69	DEP	0.50	0.83	<0.001	0.47	0.90	DEPR	0.38	0.77	DEP	0.33	0.83	<0.001	0.49	0.91
PHOB	0.14	0.29	PHO	0.00	0.25	<0.001	0.57	0.81	PHOB	0.14	0.29	PHO	0.00	0.25	<0.001	0.57	0.83	PHOB	0.00	0.43	PHO	0.00	0.25	<0.001	0.54	0.88
GSI	0.41	0.53	GSI	0.43	0.64	<0.001	0.49	0.90	GSI	0.42	0.51	GSI	0.50	0.64	<0.001	0.47	0.90	GSI	0.35	0.60	GSI	0.36	0.64	<0.001	0.51	0.93
	SCL-90-R			SCL-K-9						SCL-90-R			SCL-K-9						SCL-90-R			SCL-K-9				
GSI	0.41	0.53	GSI	0.56	0.89	<0.001	0.43	0.91	GSI	0.42	0.51	GSI	0.56	0.78	<0.001	0.41	0.91	GSI	0.35	0.60	GSI	0.50	0.78	<0.001	0.45	0.91

*SOMA* Somatization, *OCD* Obsessive-Compulsive Disorder, *INT* Interpersonal Sensitivity, *DEPR* Depression, *ANX* Anxiety, *HOST* Hostility, *PHOB* Phobic Anxiety, *PARA* Paranoid Ideation, *PSYC* Psychoticism, *GSI* Global Severity Index, *DEP* Depressive Symptoms, *DYS* Dysthymic Symptoms, *VEG* Vegetative Symptoms, *AGO* Agoraphobic Symptoms, *SOP* Symptoms of Social Phobia, *MIS* Symptoms of Mistrust

**Fig. 1:**
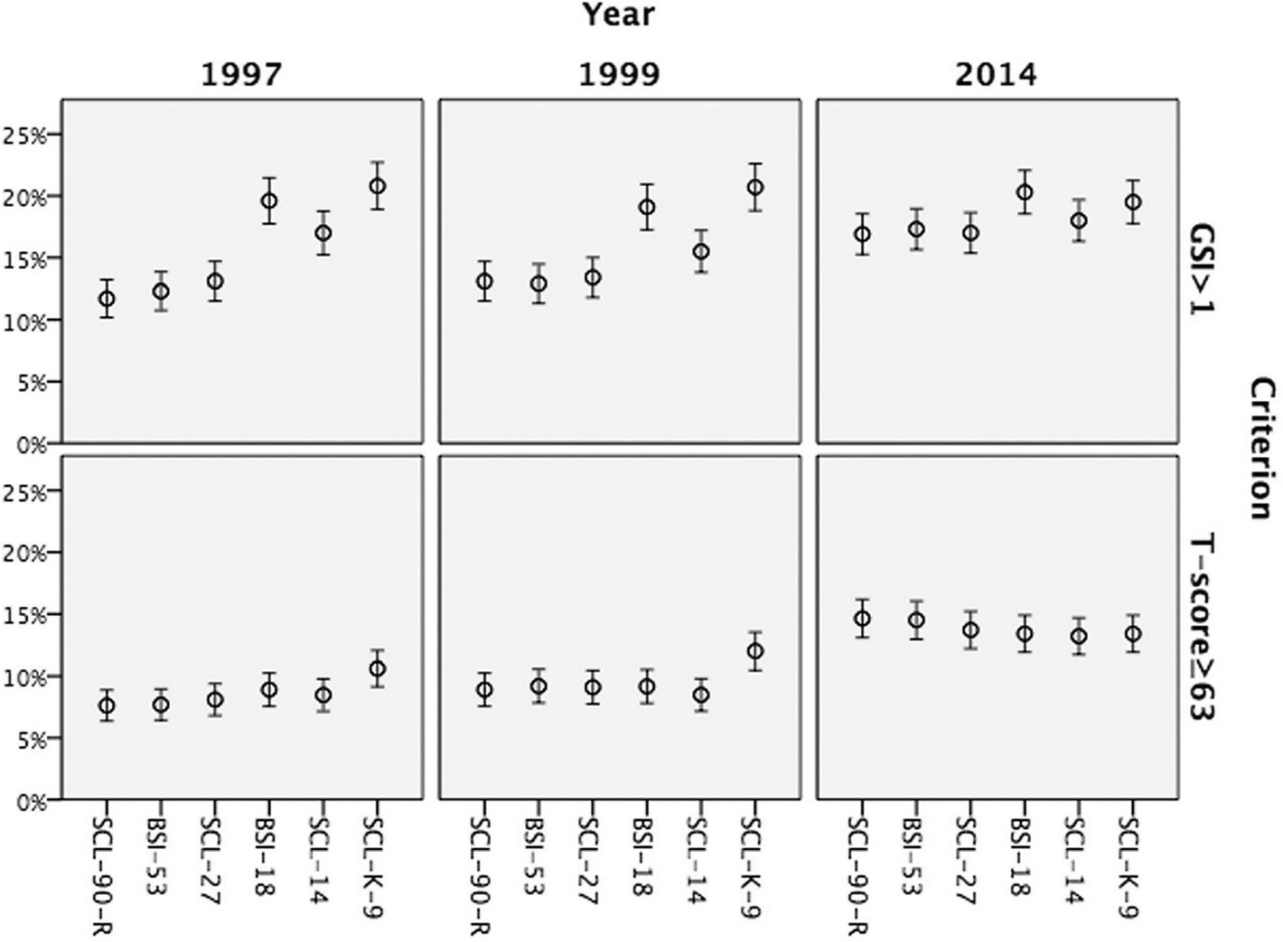
Prevalence and 95 % confidence intervals of severe symptoms in Ukraine according to different criteria (T-score≥63 and GSI>1)

When comparing full and shortened versions in the context of probable case prevalence according to Derogatis’ criterion (*T*-value for GSI ≥ 63), in 1997 and 1999 only SCL-9-K shows prevalence estimates that go beyond the 95 % confidence interval for the severe symptoms prevalence calculated by SCL-90-R (Fig. 1). In 2014, the variance of prevalence estimates in different versions of SCL-90-R is minimal.

According to the criterion of GSI > 1, the variance is greater: in 1997, 1999 and 2014 proportions of the population with severe symptoms based on BSI-18 and SCL-K-9 were significantly higher at the 5 % level than those based on SCL-90-R. In 1997, significantly higher proportions than in the full version of the SCL-90-R were also observed for SCL-14.

### Temporal stability

If we consider reliability and validity of the five versions in terms of dynamics, there is acceptable consistency of scales and model fit of latent constructs in all three studies (1997, 1999, 2014). Unfortunately, the secondary analysis does not allow us to estimate sensitivity to changes, as analyzed datasets of different years were not drawn from a single cohort.

## Discussion

Overall, our analysis supported the reliability and the original factor structure of the Ukrainian version of SCL-90-R and its five shortened versions, as well as the acceptable equivalency of selected measures.

At the level of GSI analysis it is more profitable economically to use shortened versions, particularly SCL-K-9, which consists of nine points, and in whose favor are good reliability indicators, factor validity indicators and small effect sizes. For the analysis at the level of certain symptomatic dimensions, BSI-53 offers greater opportunities, where there are fewer questions than in the SCL-90-R, but there is the same 9-dimensional structure, satisfactory reliability and factorial validity. However, the 53-symptom questionnaire is quite cumbersome for annual monitoring surveys of the general population. According to the results of this paper, the best solution would be to use BSI-18, where the factorial structure of the three symptomatic measures is confirmed, satisfactory factor model fit is observed in all three studies (1997, 1999, and 2014), and there is a good internal consistency of the subscales.

In SCL-27, the problematic aspect for Ukraine is the lack of satisfactory reliability of the depression scale. However, lower reliability indicators in certain symptomatic measurements may result from sensitivity of the Cronbach’s alpha criterion to the number of indicators within one symptomatic state [6, 37]. The scale of depressive symptoms in the SCL-27 is calculated on the basis of a small number of indicators (four symptoms).

In the previous studies the lack of factor structure confirmation was a key target of criticism; in our opinion, such results could arise not so much because of the peculiarities of the country or of the analyzed groups, but because of the limitations in the sample size and the use of irrelevant methods, such as not accounting for ordinal scales of indicators in the SCL-90-R [23, 26–28]. Ukrainian study revealed that confirmatory factor analysis on 2014 data has confirmed the structure of symptomatic scales in both full and shortened versions of the SCL-90-R. However, in the 1997 and 1999 studies, which had a smaller sample size than the 2014 study, a number of models (SCL-90-R, SCL-27, and SCL-14) have shown unsatisfactory fit according to the χ-square criterion, although on other fit indices (RMSEA, CFI, TLI) satisfactory results have been obtained.

Analysis of the equivalence of shortened and full versions of the SCL-90-R has shown the presence of small effect sizes, which is consistent with the results of validation in other countries [11, 22]. On the other hand, analysis of probable cases prevalence by different criteria (*T*-score > 63 or GSI > 1) indicates that SCL-K-9 shows higher prevalence of severe symptoms. However, the analysis of equivalence of the shortened versions and SCL-90-R for assessing the prevalence of distress requires further investigation, in particular clarification of the criteria for threshold values of probable cases in Ukraine as well as in certain population groups.

Despite a generally positive assessment of the validation of SCL-90-R as well as its shortened versions in Ukraine, a number of limitations should be noted. Firstly, SCL-90-R wasn’t the main objective of the study in any of the three analyzed surveys. The symptomatic checklist was located at the end of the monitoring questionnaire, which included a wide range of issues. This could affect the completion of the questionnaire due to fatigue of the respondents. Secondly, the 1997/1999 and 2014 studies, although they are similar in design (all three are cross-sectional and representative of the population, and used the same method of data collection), they have differences in a number of procedural aspects: sample building, a set of cluster profiles, period of field stage, and being carried out by different organizations for different objectives. Thirdly, all the periods when SCL-90-R was used in Ukraine among the general population, were characterized as periods of severe social crisis. 1997 and 1999 were characterized by significant economic difficulties, and 2014 – by the political crisis and military conflict in the east of the country, which involved a number of challenges for the whole country. There is a lack of a comparable assessment conducted during a relatively prosperous period (for example, in the 2000s before the financial crisis) that would allow for evaluation of the sensitivity of the questionnaire to such changes. Fourthly, convergent and discriminant validity of the full and shortened versions in Ukraine remain questionable as none of the appropriate alternative screening tools were used simulteniously with the SCL-90-R.

Among the strengths of our study is the fact that the results can be generalized to the entire population of Ukraine. The presence of three waves of the study at different times allowed us to check the temporal stability of the factor structure and reliability of the tool during different stages of social and economic development of the country. Another strong point of the study is the use of CFA with polychoric correlations, which allowed for improvement of the model fit. A certain advantage of the analysis is the use of non-parametric methods of analysis, in particular the description of data through the median and interquartile distance, using Spearman’s Rho correlations and Vargha and Delaney’s A effect sizes. The traditional approach to the analysis of certain symptomatic dimensions usually includes the calculation of averages, standard deviations, Pearson’s correlations and parametric effect sizes. Our study has shown that all symptomatic measurements have distributions which deviate from normal even in large samples, so the use of non-parametric methods for the validation SCL-90-R is more appropriate.

Prospects for further validation of SCL-90-R studies in Ukraine suggest evaluation of the discriminant and convergent validity using alternative questionnaires measuring psychological distress, conducting cohort studies to determine the sensitivity of the questionnaire to social changes and studying the relevant thresholds for determining probable cases of psychological distress as well as its symptomatic dimensions.

## Conclusion

This validation study of the full and shortened versions of the SCL-90-R has shown that SCL-K-9 might be an optimal solution for assessing general distress in national population monitoring studies in Ukraine. If it is necessary to analyze certain symptomatic dimensions of distress, using BSI-18 is recommended.

## Additional files

Additional file 1: Confirmatory factor analysis of the SCL-90-R in the Ukrainian general population: factor loadings. Results of confirmatory factor analysis. Factor loadings for nine dimensions of the SCL-90-R in 1997, 1999 and 2014 studies that are representative of the Ukrainian population. (XLSX 12 kb) Additional file 2: Confirmatory factor analysis of the BSI-53 in the Ukrainian general population: factor loadings. Results of confirmatory factor analysis. Factor loadings for nine dimensions of the BSI-53 in 1997, 1999 and 2014 studies that are representative of the Ukrainian population. (XLSX 11 kb) Additional file 3: Confirmatory factor analysis of the SCL-27 in the Ukrainian general population: factor loadings. Results of confirmatory factor analysis. Factor loadings for six dimensions of the SCL-27 in 1997, 1999 and 2014 studies that are representative of the Ukrainian population. (XLSX 10 kb) Additional file 4: Confirmatory factor analysis of the BSI-18 in the Ukrainian general population: factor loadings. Results of confirmatory factor analysis. Factor loadings for three dimensions of the BSI-18 in 1997, 1999 and 2014 studies that are representative of the Ukrainian population. (XLSX 9 kb) Additional file 5: Confirmatory factor analysis of the SCL-14 in the Ukrainian general population: factor loadings. Results of confirmatory factor analysis. Factor loadings for three dimensions of the SCL-14 in 1997, 1999 and 2014 studies that are representative of the Ukrainian population. (XLSX 9 kb) Additional file 6: Confirmatory factor analysis of the SCL-K-9 in the Ukrainian general population: factor loadings. Results of confirmatory factor analysis. Factor loadings for the SCL-K-9 in 1997, 1999 and 2014 studies that are representative of the Ukrainian population. (XLSX 8 kb) Additional file 7: Confirmatory factor analysis of the SCL-90-R in the Ukrainian general population: correlations between latent factors. Results of confirmatory factor analysis. Correlations between the latent variables measured by SCL-90-R items in 1997, 1999 and 2014 studies that are representative of the Ukrainian population. (XLSX 10 kb) Additional file 8: Confirmatory factor analysis of the BSI-53 in the Ukrainian general population: correlations between latent factors. Results of confirmatory factor analysis. Correlations between the latent variables measured by BSI-53 items in 1997, 1999 and 2014 studies that are representative of the Ukrainian population. (XLSX 11 kb) Additional file 9: Confirmatory factor analysis of the SCL-27 in the Ukrainian general population: correlations between latent factors. Results of confirmatory factor analysis. Correlations between the latent variables measured by SCL-27 items in 1997, 1999 and 2014 studies that are representative of the Ukrainian population. (XLSX 10 kb) Additional file 10: Confirmatory factor analysis of the BSI-18 in the Ukrainian general population: correlations between latent factors. Results of confirmatory factor analysis. Correlations between the latent variables measured by BSI-18 items in 1997, 1999 and 2014 studies that are representative of the Ukrainian population. (XLSX 9 kb) Additional file 11: Confirmatory factor analysis of the SCL-14 in the Ukrainian general population: correlations between latent factors. Results of confirmatory factor analysis. Correlations between the latent variables measured by SCL-14 items in 1997, 1999 and 2014 studies that are representative of the Ukrainian population. (XLSX 9 kb)
